# Brazilian experience with oral atenolol in the treatment of infantile hemangiomas^☆^

**DOI:** 10.1016/j.abd.2024.03.013

**Published:** 2024-11-15

**Authors:** Tauana Ogata Coelho da Rocha, Beatrice Nóbrega Dantas Berenguer, Camila Barreto Vianna Martins, Vanessa Rolim Bessa, Luciana Paula Samorano, Maria Cecília Rivitti-Machado, Zilda Najjar Prado de Oliveira

**Affiliations:** Department of Dermatology, Hospital das Clínicas, Faculty of Medicine, Universidade de São Paulo, São Paulo, SP, Brazil

*Dear Editor,*

Infantile Hemangioma (IH) affects around 5% of infants, it is the most common tumor during childhood.^1^, ^2^ At birth, lesions are usually imperceptible and develop through an early proliferative phase in the first several months of life, followed by a slower involution phase for years.^2^, ^3^

Despite being a self-limited condition in most cases, systemic treatment is indicated for IH if ulceration, bleeding, and functional, aesthetic, or even potential vital damage are present.^1^, ^3^

The first-line treatment is propranolol, however, there may be conditions that make its use unfeasible, like bronchospasm.^4^, ^5^ Atenolol, a hydrophilic cardiac beta-adrenergic receptor selective beta-blocker, with fewer potential side effects, has been studied for those cases.^5^

Sixteen patients with infantile hemangiomas treated with oral atenolol at the Department of Dermatology, Hospital das Clínicas, São Paulo University are demonstrated, displaying effective results, rapid response and a good safety profile. All presented objective indications for systemic treatment and were previously evaluated by cardiologists for safe drug intake.

The inclusion criteria considered for systemic treatment were functional impairment, presence or high risk of ulceration, life-threatening complications, or unaesthetic lesion.

Atenolol was chosen due to its easier posology (single daily administration), reduced risk of crossing the blood-brain barrier and fewer side effects compared to propranolol, given that some children were wheezing infants.

Data regarding gender, prematurity, age at the first evaluation and at the beginning of atenolol prescription, depth of the hemangioma, indication for systemic therapy, average dose used, and duration of treatment are shown in Table 1.

**Table 1:** Infantile hemangiomas treated with oral atenolol.

Case number	Age at the first evaluation (months)	Age at the first treatment day (months)	Current age (months)	Gender	Age at birth	Indication for systemic treatment	Mean dose (mg/kg/day)	Treatment duration	Treatment response
1	3m	10m	45m	Female	32 weeks	Multiple IH and hepatic IH	1.3	13m	Complete/nearly complete response
2	1m	5m	41m	Female	35 weeks	Functional impairment	1.0	17m	Complete/nearly complete response
3	11m	11m	50m	Male	Term	Unaesthetic lesion	1.1	18m^b^	Nonresponse (late introduction)
4	4m	15m^a^	39m	Male	24 weeks	Unaesthetic lesion	1.6	15m^b^	Complete/nearly complete response
5	2m	2m	35m	Female	Term	Unaesthetic lesion	1.6	17m	Complete/nearly complete response
6	5m	7m	34m	Female	Term	Risk of ulceration	1.5	11m	Complete/nearly complete response
7	9m	11m	36m	Female	Term	Risk of ulceration	1.6	9m	Partial response (late introduction)
8	2m	6m	43m	Male	27 weeks	Functional impairment	1.0	22m	Complete/nearly complete response
9	3m	4m	21m	Male	Term	Unaesthetic lesion	1.0	5m	Complete/nearly complete response
10	10m	12m	36m	Female	Term	Unaesthetic lesion	1.5	10m	Complete/nearly complete response
11	8m	9m	35m	Female	Term	Unaesthetic lesion	1.6	15m^b^	Partial response (late introduction)
12	6m	10m	35m	Female	30 weeks	Ulcerated lesion	1.5	14m	Partial response (late introduction)
13	9m	9m	48m	Female	35 weeks	Unaesthetic lesions	2.0	31m	Partial response (late introduction)
14^c^	12m	17m	40m	Female	Term	Unaesthetic lesion	2.0	19m	Complete/nearly complete response
15	3m	3m	15m	Male	Term	Unaesthetic lesion	3.0	11m	Failure
16	7m	12m	21m	Male	Term	Ulcerated lesion	1.6	9m	Partial response (late introduction)

IH, Infantile Hemangioma.

a Atenolol was indicated at seven months of life, but due to family problems, the patient started the treatment only at 15 months of age.

b Patients who temporarily stopped treatment due to the pandemic: case ‘3’ ‒ Three months, case ‘4’ – Four months and case ‘11’ ‒ Six months with no medication.

c Extensive lesion on the face ‒ excluded PHACES Syndrome.

Photographic documentation was recorded during each appointment, then analyzed and evaluated following criteria published by Pattanshetti et al.^6^ A reduction of over 90% of the lesion was considered a complete/nearly complete response, even with telangiectasia or redundant tissue (Fig. 1, Fig. 2). A partial response includes those cases with some size reduction, and color or consistency changes that did not meet the above criteria (Fig. 3).

**Fig. 1:**
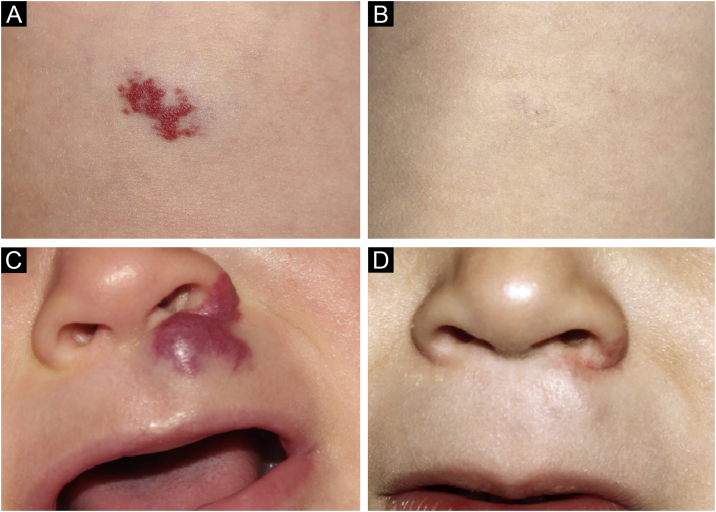
Case 5. Superficial infantile hemangioma in the right mid-axillary line. (A) Before the treatment. (B) Complete response after the treatment. Mixed infantile hemangioma on the nose. (C) Before the treatment. (D) Complete response after the treatment.

**Fig. 2:**
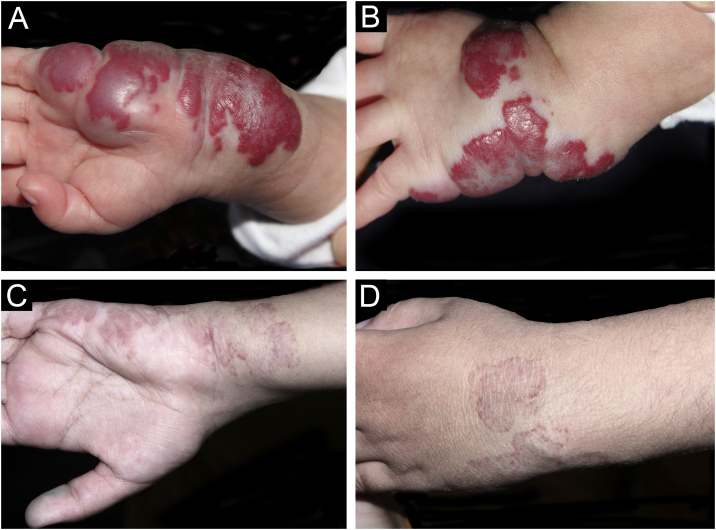
Case 8. Mixed infantile hemangioma in the left wrist and palmar region. (A) and in the back of the hand (B) , before the treatment. Palmar region (C) and back of the hand (D) with complete response after the treatment.

**Fig. 3:**
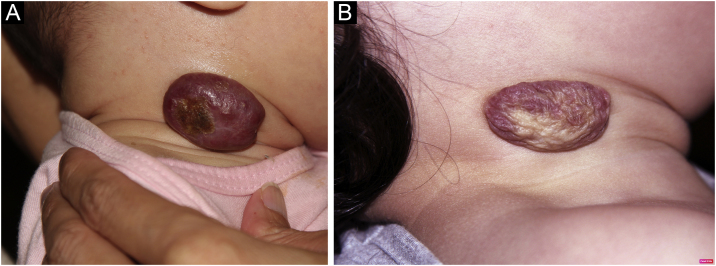
Case 12. Mixed infantile hemangioma in the right cervical region. (A) Before the treatment. (B) Partial response after the treatment.

Sixteen patients were followed up, ten girls and six boys; six patients were premature. The age of patients at the first evaluation varied between one to twelve months old (average of 5.9 months), while age at the beginning of treatment ranged from two to seventeen months (average of 8.9 months). Twenty-two lesions on sixteen patients were evaluated; three deep hemangiomas (13.6%), nine superficial (40.9%), and ten mixed (45.4%). One had multiple cutaneous infantile hemangiomas associated with hepatic hemangiomas.

Systemic treatment was due to aesthetic disfigurement in ten patients (62%), nine with face lesions and one on the neck, followed by ulcerated hemangioma or high risk of ulceration in four patients (25%) and risk of functional impairment in two cases (12.5%). Doses ranged from 1.0 to 3.0 mg/kg/day and doses over the average of 1.5 mg/kg/day were indicated to those with no initial response or potential seriously complicated IHs.

The treatment lasted between five and thirty-one months. The shortest treatment was related to a complete clinical and ultrasonographic response. On the other hand, the longest case had a late drug introduction, at the age of nine months and atenolol was suspended at the age of 24 months, but regrowth of the HI led to reintroduction during a seven-month course, leading to a partial response.

The patient with multiple cutaneous infantile hemangiomas associated with hepatic lesions had her lesions completely resolved by eighteen months, the hepatic lesions regressed faster than cutaneous.

No side effects were observed.

Infantile hemangioma is the most common vascular tumor during childhood, with a higher prevalence among females and Caucasians.^7^ Propranolol, a lipophilic nonselective beta-blocker, has been the first line therapy. Although presenting a good safety profile, side effects such as sleep disturbance, bradycardia, bronchial reactivity, and hypoglycemia may be a limiting factor for some patients.^5^

Atenolol, a selective β1-adrenergic receptor blocking agent, has been used as an alternative to propranolol. Due to its selectivity, the risk of hypoglycemia and bronchospasm is reduced; being less lipophilic than propranolol, it does not cross the blood-brain barrier, making sleep disorders unlikely.^5^ Atenolol can be taken in a single daily dose, which improves adherence to treatment.^8^ The target dose is 1 mg/kg/day, but, in cases of partial response or late introduction, it can be used up to 3 mg/kg/day.^4^, ^5^

In 2014, Ábarzúa-Araya et al.^9^ reported the first randomized clinical trial comparing the efficacy of propranolol and atenolol for IHs, however the sample size of the study was not enough for statistical power. In 2021, Yi Ji et al.^10^ conducted a new randomized multicenter clinical trial comparing these two β-blockers, demonstrating similar response rates: 93.7% in the propranolol group and 92.5% in atenolol group.

In our study, the response was observed in fourteen cases (87.5%); there was complete regression in 9 cases (56.25%) and partial in 5 (31.25%); no improvement was noted in 2 (12.5%). Partial response may be attributed to the late introduction of atenolol, after the proliferative phase; the same occurred in one of the non-responders. The other one may be considered a treatment failure, as atenolol was timely introduced and higher doses were used (3 mg/kg/day).

This is the first Brazilian report on the use of atenolol to treat problematic IHs in a tertiary service. Most cases (87.5%) responded to the treatment. A better response was observed when treatment started in the proliferative phase. There was one primary failure (12.5%) and no side effects were noted.

Of note, treatment was often initiated after the proliferative phase of IH due to late access to a proper diagnosis and treatment; even so, those cases responded well, another evidence of the efficacy of the drug. Treatment was well tolerated, including by wheezing infants. In conclusion, atenolol was found a safe, effective, and well-tolerated alternative for the treatment of infantile hemangiomas.

## Financial support

None declared.

## Authors’ contributions

Tauana Ogata Coelho da Rocha: Design and planning of the study; drafting and editing of the manuscript; critical review of the literature.

Zilda Najjar Prado de Oliveira: Approval of the final version of the manuscript.

Luciana Paula Samorano: Critical review of the intellectual content; effective participation in research orientation.

Maria Cecília Rivitti-Machado: Critical review of the intellectual content; effective participation in research orientation.

Camila Barreto Vianna Martins: Collection of data; critical review of the literature.

Vanessa Rolim Bessa: Collection of data; critical review of the intellectual content.

## Conflicts of interest

None declared.
